# DHCR24 associates strongly with the endoplasmic reticulum beyond predicted membrane domains: implications for the activities of this multi-functional enzyme

**DOI:** 10.1042/BSR20130127

**Published:** 2014-03-18

**Authors:** Eser J. Zerenturk, Laura J. Sharpe, Andrew J. Brown

**Affiliations:** *School of Biotechnology and Biomolecular Sciences, The University of New South Wales, Sydney, NSW 2052, Australia

**Keywords:** cholesterol, DHCR24, ER, membrane, peduncle, topology, CHO, Chinese-hamster ovary, DAPI, 4′,6-diamidino-2-phenylindole, DHCR24, 3β-hydroxysterol Δ24-reductase, ER, endoplasmic reticulum, FAD, flavin adenine dinucleotide, GRAVY, grand average of hydropathicity, HMM, hidden Markov model, MSA, multiple sequence alignment, PTM, post-translational modification, SA, signal anchor, SP, signal peptide, TM, transmembrane, TMD, transmembrane domain

## Abstract

Cholesterol synthesis occurs in the ER (endoplasmic reticulum), where most of the cholesterogenic machinery resides. As membrane-bound proteins, their topology is difficult to determine, and thus their structures are largely unknown. To help resolve this, we focused on the final enzyme in cholesterol synthesis, DHCR24 (3β-hydroxysterol Δ24-reductase). Prediction programmes and previous studies have shown conflicting results regarding which regions of DHCR24 are associated with the membrane, although there was general agreement that this was limited to only the N-terminal portion. Here, we present biochemical evidence that in fact the majority of the enzyme is associated with the ER membrane. This has important consequences for the many functions attributed to DHCR24. In particular, those that suggest DHCR24 alters its localization within the cell should be reassessed in light of this new information. Moreover, we propose that the expanding database of post-translational modifications will be a valuable resource for mapping the topology of membrane-associated proteins, such as DHCR24, that is, flagging cytosolic residues accessible to modifying enzymes such as kinases and ubiquitin ligases.

## Abstract 2

We present new findings indicating that the final enzyme in cholesterol synthesis is strongly associated with the endoplasmic reticulum membrane throughout its length, contrary to previous studies that indicated only the beginning of the enzyme was associated with the membrane.

## INTRODUCTION

Cholesterol synthesis enzymes are typically localized to the ER (endoplasmic reticulum), and as membrane-associated proteins, little has been done to determine their exact topology and structure. We are particularly interested in DHCR24 (3β-hydroxysterol Δ24-reductase), the final enzyme in cholesterol synthesis, as it is a multifunctional protein, which is intricately regulated (reviewed in [1]). Initial studies characterizing DHCR24 by Greeve et al. [2], and subsequently confirmed by Wu et al. [3], found that it was targeted to the ER, and to a lesser degree, to the Golgi.

Since the discovery of DHCR24, numerous binding partners and cofactors have been identified and characterized. DHCR24 contains a highly conserved FAD (flavin adenine dinucleotide)-binding domain [4–6], and reduction of desmosterol to cholesterol is dependent on FAD [7], suggesting functionality of the conserved domain. In addition, DHCR24 contains conserved binding domains for both p53 and Mdm2, required for mediating cellular responses to oncogenic and oxidative stress [3]. DHCR24 is reported to be proteolytically cleaved during apoptosis, at two highly conserved caspase recognition motifs located in the FAD- and p53-binding domains, presumably destroying them, producing a soluble, 40 kDa peptide [2]. How DHCR24 interacts with the membrane, however, is less well known.

Using simulated DHCR24 membrane models with and without substrate and cofactors, Pedretti et al. [5] predicted DHCR24 as a monotopic membrane protein; with the N-terminus partially embedded in the membrane as a stem or ‘peduncle’ (meaning ‘little foot’), rather than traversing the membrane bilayer (bitopic). This ‘peduncle’ firmly anchors the protein to the membrane, with the C-terminus protruding into the cytoplasm, allowing access to substrates and cofactors [5]. The DHCR24 homologue, DWF1 in *Arabidopsis thaliana*, has a similar predicted structure, based on hydropathy predictions: strong membrane association and a cytoplasmic C-terminus [8].

However, Lu et al. [9] published the experimental work in support of a bitopic, single TMD (transmembrane domain) model of DHCR24: a lumenal N-terminus, followed by a single TMD (also at the N-terminus), and cytoplasmic C-terminus. The N-terminus was essential for DHCR24 membrane association, as deletion of this region (1–58, Δ58 DHCR24) translocated DHCR24 to the cytoplasm. The orientation of the N- and C-termini were deduced from protease protection assays of fluorescent fusion constructs; an N-terminal Ds-Red fluorescent fusion protein was preserved, indicating that it was lumenal, whereas a C-terminal green fluorescent protein was degraded by trypsin, indicating cytoplasmic localization. By contrast, Pedretti et al.'s ‘peduncle’ model [5] predicts that the N-terminus is buried in the membrane, and the C-terminus is cytoplasmic. However, fusion of the large (~28 kDa), soluble, Ds-Red fluorescent protein to the N-terminus could feasibly draw the membrane-embedded N-terminus into the lumen, that is, interfere with the native membrane topology of DHCR24. Therefore we aimed to determine the validity of the two different published models [5,9], investigating how DHCR24 interacts with the ER membrane, and its membrane topology.

## MATERIALS AND METHODS

### Materials

CHO (Chinese-hamster ovary)-7 cells, the Insig-myc plasmid and the antibody against the trypsin-protected SREBP-cleavage activating protein (Scap, IgG-R139) fragment were generous gifts from Drs Michael S. Brown and Joseph L. Goldstein (UT Southwestern Medical Center, Dallas, TX, USA). IgG-R139 is a rabbit polyclonal antibody against hamster Scap (residues 54–277 and 540–707 [10]). Trypsin was from Sigma-Aldrich. Lipofectamine LTX Reagent was from Life Technologies. Plasmids expressing DHCR24 with a V5 epitope tag at the N- or C-terminus were created in pcDNA3.1 (Life Technologies). Truncations of the DHCR24-V5 plasmid were made using PIPE (polymerase incomplete primer extension) [11] or by two stage mutagenesis [12] for Δ56 DHCR24-V5.

### Cell culture and transfection

CHO-7 cells were cultured in 5% (v/v) LPDS (lipoprotein-deficient serum)/Dulbecco's modified Eagle's medium/Ham's F12 (DMEM/F12). Cells were transfected using Lipofectamine LTX Reagent according to the manufacturer's instructions.

### Cell fractionation

The cell fractionation protocol was adapted from Feramisco et al. [13]. Cells were washed and scraped into PBS on ice and centrifuged at 1000 ***g***. The cellular pellet was resuspended in 400 μl Buffer A [10 mM Hepes–KOH (pH 7.4), 10 mM KCl, 1.5 mM MgCl_2_, 5 mM sodium EDTA, 5 mM sodium EGTA and 250 mM sucrose] and passed through an 18 gauge needle 50 times. Protein concentration of cell lysate was determined using the BCA assay. Cell lysate was centrifuged at 1000 ***g*** for 5 min at 4°C. The 1000 ***g*** pellet was resuspended in 100 μl Buffer B [20 mM Hepes–KOH (pH 7.6), 25% (v/v) glycerol, 0.42 M NaCl, 1.5 mM MgCl_2_, 5 mM sodium EDTA, 5 mM sodium EGTA], and incubated at 4°C for 1 h with end over end mixing, and centrifuged at 100000 ***g*** for 30 min at 4°C. The supernatant from this centrifugation was collected and designated the nuclear fraction.

The supernatant from the 1000 ***g*** centrifugation was centrifuged at 100000 ***g*** for 30 min at 4°C. The supernatant was collected and designated the cytosolic fraction. The pellet was resuspended in 100 μl Buffer C [10 mM Tris–HC1 (pH 7.4), 100 mM NaC1, 1% (w/v) SDS], and designated the membrane fraction.

### Membrane isolation

To prepare membranes for differential solubilization and protease protection assays, cells were washed and scraped in PBS and centrifuged at 1000 ***g*** for 5 min at 4°C. The cellular pellet was resuspended in Buffer A or D (Buffer A containing 100 mM NaCl; as indicated in figure legends), passed through an 18 gauge needle 50 times, and the cell lysate was centrifuged at 1000 ***g*** for 5 min at 4°C. The supernatant was then centrifuged at 20000 ***g*** for 15 min at 4°C, and the resulting membrane pellet was resuspended in 65 μl Buffer A or D. Membrane protein concentration was determined using the BCA assay.

### Differential solubilization

To determine the strength of the protein–membrane interaction, equivalent amounts of membrane were treated with 200 μl 1% SDS, Buffer A, 0.1 M Na_2_CO_3_ (pH 11.5), or 1 M NaCl, and incubated at 4°C for 30 min with end over end mixing. Treated membranes were centrifuged at 100000 ***g*** for 30 min at 4°C; the supernatant was designated the soluble cytoplasmic fraction (C), and the membrane pellet was resuspended in 200 μl Buffer D and designated the insoluble membrane fraction (M).

### Protease protection assay

To determine the membrane orientation of the N- and C-termini, equivalent amounts of membrane were treated with trypsin as described [13]. Briefly, membranes were treated with the indicated amount of trypsin in the presence or absence of Triton X-100 in Buffer A, for 30 min at 30°C. Reactions were stopped by the addition of loading buffer and heat inactivation at 95°C for 10 min.

### Immunofluorescence microscopy

To determine intracellular localization, cells grown on coverslips were cotransfected with a DHCR24 expression construct and an ER marker plasmid (pDsRed-ER, Clontech). Cells were then fixed with 3% (v/v) formaldehyde/PBS for 10 min. Cells were rinsed with PBS (three times for 5 min) and then permeabilized with 0.3% (v/v) Triton X-100/PBS for 5 min. Cells were washed with PBS, then incubated with 10% (v/v) FCS/PBS for 1 h. Cells were then incubated with 1:2000 V5 antibody in 10% FCS/PBS including 0.1% (w/v) saponin for 16 h at 4°C. After washing with 10% FCS/PBS (three times for 5 min), cells were incubated with 1:1,000 Alexa Fluor-488-conjugated secondary antibody (Molecular Probes) in 10% FCS/PBS for 1 h. Cells were washed with 10% FCS/PBS (three times for 5 min) and then mounted on glass slides with ProLong Gold AntiFade Reagent with DAPI (4′,6-diamidino-2-phenylindole; Life Technologies). Images were obtained using a Nikon C1 confocal microscope with laser excitation at 408 nm (DAPI), 488 nm (DHCR24-V5) and 561 nm (ER marker).

### Bioinformatics tools

For *in silico* analysis, the complete human amino acid sequence of DHCR24 (Q15392) was used. An SP (signal peptide) was predicted using SignalP v4.1 [14] and a hydrophobicity profile was returned by ProtScale [15]. TMDs and membrane topology were predicted using TMHMM (TM hidden Markov models) 2.0 [16], TOPCONS [16] and ΔG predictor server v1.0 [17]. Myristoylator [18], NMT [19], big-PI predictor [20] PrePS [21], CSS-Palm 2.0 [22] were used to predict PTMs (post-translational modifications) involved in membrane attachment. To model the membrane topology, the LaTeX package TeXtopo [23] was employed, with a minor modification to the source code to enable a ‘half-loop’ to extend beyond 14 amino acids.

## RESULTS

### DHCR24 has an extremely hydrophobic N-terminus, suggesting a candidate region for TMDs

**Figure 1:**
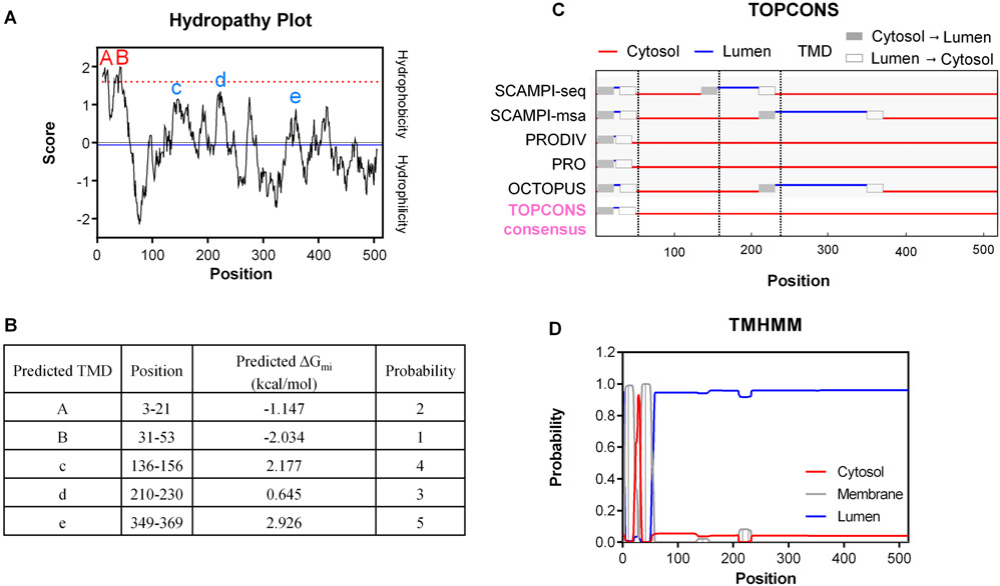
DHCR24 TMD and membrane topology predictions (**A**) A hydropathy profile of DHCR24 using the method of Kyte and Doolittle [15], returned by ProtScale with a window size of 19. The recommended threshold for the detection of TMDs is indicated by the dotted red line (1.6) [15], with peak regions of hydrophobicity above this marked in red (A and B). The GRAVY score is indicated by the solid blue line (−0.061). Predicted TMDs based on the apparent free energy difference for ER membrane insertion by the Sec61 translocon (ΔG_mi_) below the 1.6 threshold are marked in blue (c, d, e) (**B**) Putative TMDs and their probability based on ΔG_mi_ [24]. (**C**) TMD predictions by the five TOPCONS algorithms: SCAMPI-seq and SCAMPI-msa [25], PRODIV and PRO [26] and OCTOPUS [27] and the TOPCONS global prediction (TOPCONS consensus) [25]. Potential TMDs are boxed grey (cytosol → lumen) or white (lumen → cytosol). Dotted grey lines indicate DHCR24 truncations, which are used in Figures 3–5. (**D**) DHCR24 membrane topology as predicted by the TMHMM server [16], with the probability of cytosol, membrane or lumenal location in red, grey or blue, respectively.

To investigate the topology of DHCR24, we first used an *in silico* approach. TM (transmembrane) proteins are hydrophobic by nature, and can therefore be predicted based on this characteristic. The human DHCR24 sequence from UniProt (Q15392) was analysed for putative TMDs based on hydrophobicity by the method of Kyte and Doolittle (Figure 1A) [15]. Two potential TMDs were identified at peak regions of hydrophobicity at the extreme N-terminus of DHCR24 (A, B; Figure 1A), with scores above the recommended threshold by Kyte and Doolittle (dotted red line, 1.6) [15]. As DHCR24 is not an extremely hydrophobic protein overall, having a GRAVY (grand average of hydropathicity) score of −0.061 (blue line), other hydrophobic peaks are discernible above this value, but do not reach the Kyte and Doolittle threshold of 1.6.

A more specific prediction of regions that lie within the ER membrane is the free energy calculation for membrane insertion by means of the Sec61 translocon (ΔG_mi_): negative ΔG_mi_ values are indicative of potential TMDs of strong hydrophobicity and sufficient length; positive ΔG_mi_ values are less likely to be TMDs [24]. Both potential TMDs identified by the method of Kyte and Doolittle have a negative ΔG_mi_, further indicating them as putative TMDs (A, B; Figure 1B). Three additional regions were identified as putative TMDs based on their ΔG_mi_ values (c, d, e; Figures 1A and 1B); however, their ΔG_mi_ values were positive, and these regions did not reach the threshold for detection of TMDs in the hydropathy plot. Therefore based on their hydrophobicity and their ΔG_mi_ values, they are less likely to form TMDs.

### DHCR24 is not predicted to associate with the membrane via post-translational modifications

Hydrophobic PTMs (post-translational modifications) can facilitate membrane attachment. Common groups include the irreversible attachment of various lipid groups, such as prenyl, myristoyl or GPI (glycophosphatidylinositol) groups or the reversible attachment of a palmitoyl group. The prediction programs Myristoylator [18], NMT [19], big-PI predictor [20], PrePS [21] and CSS-Palm 2.0 [22] predict that DHCR24 is unlikely to associate with the ER membrane via PTMs; therefore, these means of membrane attachment were not considered for DHCR24.

### Predicted membrane topology of DHCR24 consists of N-terminal TMDs

For advanced DHCR24 TMD prediction, TOPCONS and TMHMM membrane topology prediction programs were employed. Both of these programs use HMM and/or neural network algorithms to integrate sequence information, and MSAs (multiple sequence alignments). TOPCONS uses multiple TMD prediction programs: SCAMPI-seq and SCAMPI-msa [25], PRODIV and PRO [26] and OCTOPUS [27]. The TOPCONS global prediction (TOPCONS consensus) [25] is a consensus of these five predictions, as well as the ΔG_mi_ [24] and ZPRED [28] algorithms. Both regions identified by their hydrophobicity and ΔG_mi_ as putative TMDs (A, B; Figure 1A) and the three regions with positive ΔG_mi_ values (c, d, e; Figure 1A) were predicted by at least one of the TOPCONS prediction algorithms (Figure 1C); however, only the two N-terminal hydrophobic regions (A, B) were uniformly predicted by all five of the individual algorithms, and the global TOPCONS prediction (TOPCONS consensus; Figure 1C). Furthermore, the self-assessed reliability of the TOPCONS consensus is lowest in the region of the three other putative TMDs (c, d, e).

The TMHMM prediction [16], which also includes information on evolutionary conservation from MSAs, agreed with the TOPCONS consensus, predicting two N-terminal TMDs with high probability (Figure 1D). However, TOPCONS predicted a cytosolic C-terminus, whereas TMHMM predicted it to be lumenal.

### A secretory SP is predicted for DHCR24

DHCR24 contains a putative TMD at its extreme N-terminus (A; Figure 1), which is usually the location of a secretory sequence. Signal sequences allow translocation of a protein to, or across, the ER membrane, which is cleaved for targeting beyond the ER membrane (SP), or retained within the membrane for ER membrane localization [SA (signal anchor)] [29]. Both SPs and SAs contain a stretch of hydrophobic residues, and therefore can be incorrectly predicted as TMDs by many prediction algorithms [30]. Most TMD prediction programs, such as TMHMM cannot discriminate between N-terminal TMDs and SPs and SAs [30]. Prediction algorithms, such as SignalP, use a neural network to distinguish N-terminal TMDs from SPs based on a combined scoring system for each position at the N-terminus [31]. The most N-terminal putative TMD for DHCR24 (A; Figure 1A) was predicted to be an SP (Supplementary Figure S1; available at http://www.bioscirep.org/bsr/034/bsr034e098add.htm), with the cleavage site at 22–23 based on all scoring systems: predicted cleavage site (C-score), SP length (S-score), and the combined cleavage site score (Y-score; a combination of the C-score and the slope of the S-score), which is a better cleavage site prediction than the raw C-score alone.

To test whether the putative SP is required for ER membrane localization, truncated DHCR24 (Δ23 DHCR24) was transfected into CHO-7 cells, and the cellular localization examined. Through cell fractionation, Δ23 DHCR24 was smaller than WT DHCR24 and, like WT DHCR24, Δ23 DHCR24 localized primarily to the membrane fraction (results not shown). This demonstrates that the putative SP is not cleaved off in WT DHCR24, and is not necessary for membrane retention.

The discounted SP and TMD lie in one of the least conserved regions of DHCR24. The C-terminal region, however, is highly conserved and contains multiple protein-binding/recognition sites for FAD [4–6], caspase 3 [2], p53 and Mdm2 [3]. The three predicted TMDs that did not have negative ΔG_mi_ values all reside close to functional domains of DHCR24 (136–156, FAD; 210–230, Mdm2; 349–369, p53), that by definition should be cytosolic. Furthermore, a single TMD model is fitting, as it would allow the C-terminus to reside in the cytoplasm, making the multiple binding sites in DHCR24 accessible to soluble-binding partners.

### Membrane orientation of the N and C termini of DHCR24

**Figure 2:**
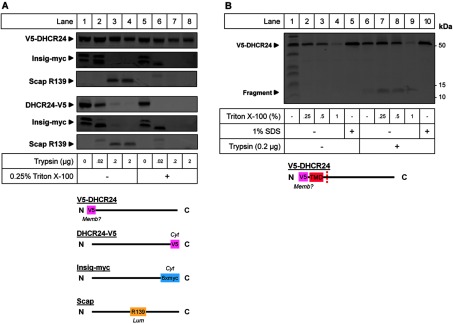
Membrane orientation of the N- and C-termini of DHCR24 CHO-7 cells were transfected with 4 μg V5-DHCR24 or DHCR24-V5, and co-transfected with 1 μg Insig-myc in a 10-cm dish for 24 h. (**A**) Cell lysate was fractionated and membranes were isolated and digested with the indicated amount of trypsin in the presence or absence of Triton X-100. (**B**) Cell lysate was fractionated and membranes were isolated and treated with Triton X-100 or 1% (w/v) SDS in the presence or absence of trypsin as indicated. Membranes were separated by SDS–PAGE (10% gel) and immunoblotted with antibodies against the V5 epitope for DHCR24, myc epitope for Insig and R139 epitope for Scap. A protein standard was added to the sample in lane 1. Data from at least *n*=2 experiments. Schematics of (**A**) V5-DHCR24, DHCR24-V5, Insig-myc and Scap with the membrane orientation of epitopes and (**B**) V5-DHCR24 with the membrane orientation of the V5 epitope and trypsin cleavage site (dotted red line) indicated in relation to the putative TMD are provided below the corresponding immunoblots.

To determine the localization of the N- and C-termini of DHCR24 with respect to the ER membrane, CHO-7 cells were transfected with DHCR24 with a V5 epitope tag at either the N-terminus (V5-DHCR24) or C-terminus (DHCR24-V5), and co-transfected with Insig, an integral membrane protein with a myc epitope on the cytoplasmic C-terminus (Insig-myc) [13] (Figure 2A). Intact ER membrane vesicles were isolated and treated with trypsin in the presence or absence of Triton X-100, subjected to SDS–PAGE and immunoblotted for V5 (Figure 2A). As controls, samples were also immunoblotted with myc for Insig, and R139 for Scap, which recognizes a lumenal loop (residues 540–707) in hamster Scap [32] (Figure 2A). Therefore when trypsin was added to microsomes, the myc epitope on Insig was digested, and the R139 epitope of Scap remained intact, as it is located within the lumen (Figure 2A, lanes 1–4). Similar to Insig, DHCR24-V5 was digested. However, V5-DHCR24 remained stable (Figure 2A, lanes 1–4). When Triton X-100 was added to partially solubilize the membrane, allowing trypsin access to lumenal peptides, the R139 epitope of Scap was digested. However, V5-DHCR24 remained relatively stable (Figure 2A, lanes 5–8), indicating that the N-terminus was protected from trypsin digestion by the membrane.

Although there was no obvious change in the size of the V5-DHCR24 band with trypsin and Triton X-100 treatment, the band intensity slightly decreased (Figure 2A, lanes 5–8), and a small fragment was discernible with increasing amounts of Triton X-100 (~11 kDa, Figure 2B, lanes 6–9). This smaller band was not visible in non-treated conditions (lanes 1–4) or when trypsin was inactivated with SDS (lane 10). Furthermore, the band appeared with or without Triton X-100 treatment, indicating a partially accessible cytoplasmic loop following a membrane embedded N-terminus. The calculated molecular weight of this digested fragment indicates that the loop is located between residues 70–100, which contains six possible arginines/lysines that could be cleaved by trypsin (dotted red line; Figure 2B). Altogether, these data are indicative of a cytoplasmic C-terminus and a non-accessible N-terminus, which is either embedded within the membrane, or lumenal but inaccessible, due to strong interactions with the ER membrane (Figure 2).

### The putative TMD is not essential for DHCR24 membrane association

**Figure 3:**
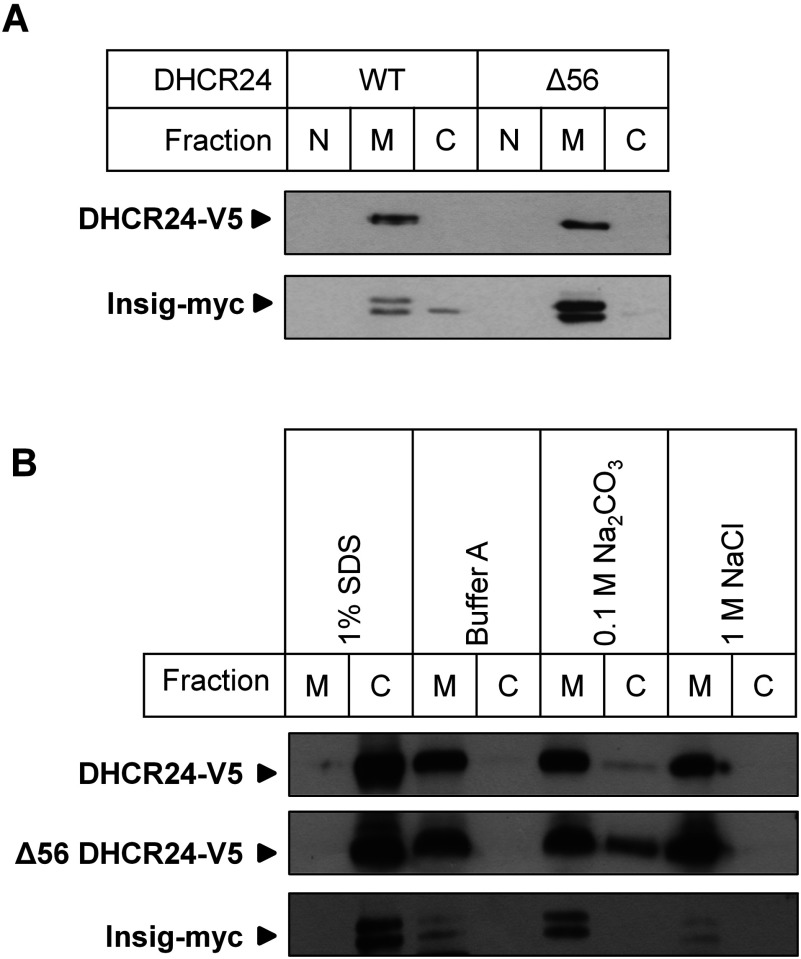
Membrane association of DHCR24 and Δ56 DHCR24 CHO-7 cells were transfected with either 8 μg DHCR24-V5 (WT) or Δ56 DHCR24-V5 (Δ56), and co-transfected with 2 μg Insig-myc in a 14.5-cm dish for 24 h. (**A**) Cell lysate was fractionated: nucleus (N), membrane (M) and cytoplasm (C). (**B**) Cell lysate was fractionated and membranes were isolated and resuspended in 1% (w/v) SDS (strong detergent), Buffer A (aqueous buffer), 0.1 M Na_2_CO_3_ pH 11.5 (high pH) or 1 M NaCl (high salt), and incubated for 30 min at 4°C with end over end mixing, then ultra-centrifuged at 100 000×***g*** and the supernatant, representing cytoplasm (C) and pellet, representing membrane (M) were collected. Cellular fractions were separated by SDS–PAGE (7.5% gel) and immunoblotted with antibodies against V5 (DHCR24) and myc (Insig). Data from at least *n*=2 experiments.

To determine if the hydrophobic N-terminus containing the putative TMD of DHCR24 is required for ER membrane attachment, truncated DHCR24 (Δ56) was transfected into CHO-7 cells, and the cellular localization examined. DHCR24-V5, like Insig-myc, localized only to membranes (Figure 3A). The truncation lacking the putative TMD, Δ56 DHCR24-V5, also localized specifically to membranes (Figure 3A). Therefore contrary to the predictions in Figure 1, these very hydrophobic regions are not necessary for DHCR24 membrane association.

### DHCR24 associates strongly with membranes

To determine if DHCR24 is an integral membrane protein, differential solubilization was performed on membranes to determine the manner in which DHCR24 associates with it. The ability of buffers (detergent, high pH, high salt) to dissociate DHCR24 from the membrane (M) into the cytoplasm (C) indicates the strength of its association with the membrane (ionic or hydrophobic).

When exposed to aqueous buffer, DHCR24 was located in the membrane fraction, but was released into the cytoplasmic fraction upon treatment with 1% (w/v) SDS (Figure 3B). Treatment with 0.1 M Na_2_CO_3_ or 1 M NaCl, reagents known to disrupt peripheral associations by altering pH and electrostatic interactions, respectively, were not able to solubilize DHCR24, and it remained in the membrane fraction (Figure 3B). For all treatments, Insig-1 displayed similar results, indicating DHCR24 is associated with membranes, and that it is an integral membrane protein (Figure 3B). Similarly, Δ56 DHCR24-V5 behaved like an integral membrane protein (Figure 3B). Although Δ56 DHCR24-V5 remained in the membrane fraction after treatment with 0.1 M Na_2_CO_3_, the presence of a weaker signal in the cytoplasmic fraction indicated that Δ56 might be solubilized with this treatment. However, performing the treatment and centrifugation process a second time showed that Δ56 DHCR24-V5 remained membrane associated (Supplementary Figure S2; available at http://www.bioscirep.org/bsr/034/bsr034e098add.htm), suggesting that regions beyond the first 56 residues are integral for strong membrane association.

### Other candidate TMDs are not essential for DHCR24 membrane association

**Figure 4:**
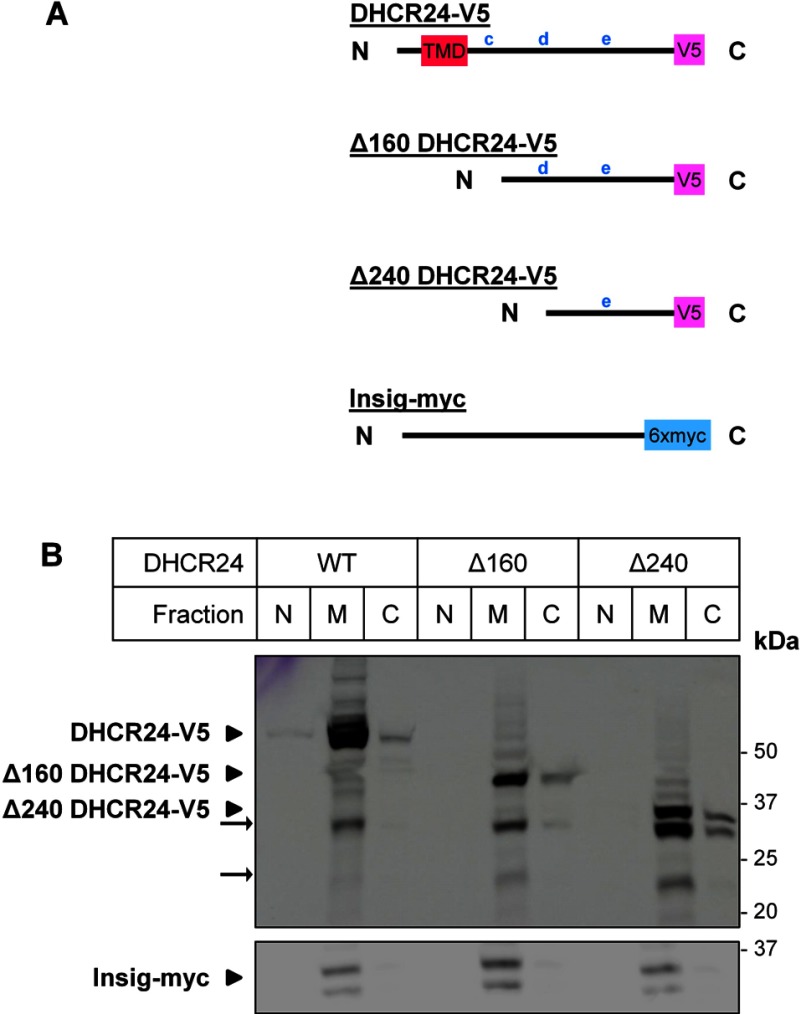
Membrane association of DHCR24 N-terminal truncations (**A**) The schematics of DHCR24-V5, Δ160 DHCR24-V5 and Δ240 DHCR24-V5 shown in relation to the putative TMD and other lower scoring putative TMDs (c, d, e) and Insig-myc. (**B**) CHO-7 cells were transfected with either 4 μg DHCR24-V5 (WT), Δ160 DHCR24-V5 (Δ160) or Δ240 DHCR24-V5 (Δ240), and co-transfected with 1 μg Insig-1-myc in a 10-cm dish for 24 h. Cell lysate was fractionated, and the nuclear (N), membrane (M) and cytoplasmic (C) fractions were separated by SDS–PAGE (10% gel) and immunoblotted with antibodies against V5 (DHCR24) and myc (Insig). Data from *n*=3 experiments.

**Figure 5:**
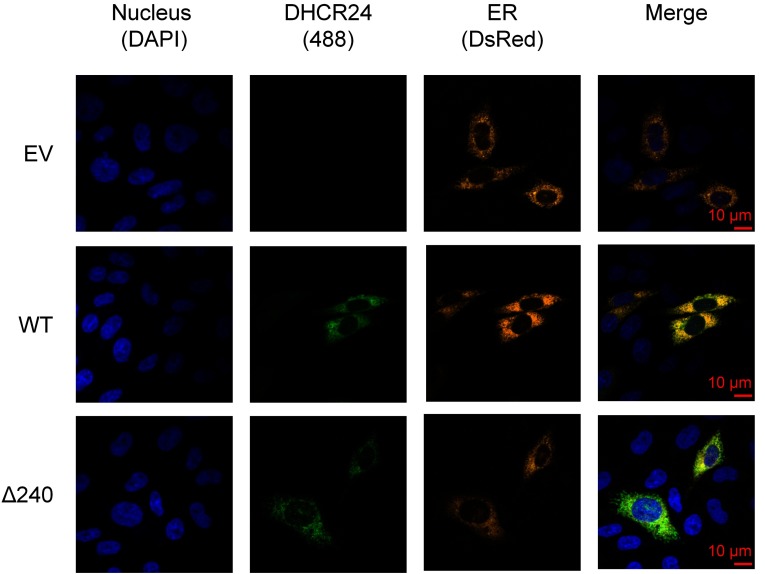
ER localization of DHCR24 and Δ240 DHCR24 CHO-7 cells were transfected with 1 μg EV (empty vector), DHCR24-V5 (WT) or Δ240 DHCR24-V5 (Δ240) and 0.25 μg pDsRed-ER in a 12-well plate for 24 h. Cells were subjected to immunofluorescence with V5 antibody and Alexa Fluor 488, then mounted on slides with DAPI-containing mounting buffer. Fluorescence microscopy was performed. Data from *n*=2 experiments.

Having established that the most likely TMDs (A and B, Figure 1) was not essential for membrane localization, we next examined the other predicted TMDs. Based on the ΔG_mi_ values, and the TMHMM probability score (Figure 1), the two next best TMD candidates were examined (c, 136–156; d, 210–230). Truncated versions of DHCR24 (Δ160, Δ240; Figure 4A) were transfected into CHO-7 cells, and the cellular localization examined. Similar to WT DHCR24–V5, both truncations were found primarily in the membrane fraction (Figure 4B), demonstrating that the first 240 residues are also not necessary for membrane attachment. The appearance of secondary bands (~22 and ~32 kDa) in the Δ240 DHCR24 membrane fraction is indicative of proteolytic fragments, which are also associated with membranes, and would encompass the untested putative TMD (e). This further suggests that there are more membrane-binding site(s) closer to the C-terminus. To confirm that the truncations are not mislocalized or aggregating, immunofluorescence microscopy was performed. WT and Δ240 DHCR24 both localized predominantly to the ER (Figure 5), indicating that DHCR24 remains ER membrane associated despite N-terminal deletions.

## DISCUSSION

To further elucidate the membrane topology of DHCR24, we predicted how it interacts with the ER membrane through the use of numerous membrane insertion algorithms, as well as considering the current literature on DHCR24 topology. We then characterized this interaction using biochemical methods.

To create a DHCR24 topology model, we first characterized DHCR24 based on its hydropathy using the method of Kyte and Doolittle [15]. Two regions of peak hydrophobicity were identified as candidates for TMDs (Figure 1A), which were confirmed by numerous TMD prediction algorithms (ΔG_mi_; Figure 1B) and programs (TOPCONS and TMHMM; Figures 1C and 1D). DHCR24 was predicted to contain an SP (Supplementary Figure S1), which generally targets proteins to or across the ER membrane, and this was located at the first putative TMD. The efficacy of a DHCR24 SP was tested by the creation of a truncation construct, which did not contain the putative SP. This was smaller than its WT DHCR24 counterpart, and still associated with membranes. Secondly, the N-terminal V5 tagged DHCR24 construct (V5-DHCR24) was the same size as C-terminal DHCR24-V5 (V5-tagged DHCR24), indicating no cleavage of the N-terminus, which would be predicted for a true SP. These data show that the N-terminus is not cleaved, nor is it required for membrane targeting, demonstrating that DHCR24 does not contain an N-terminal SP. This is similar to cytochrome P450, which is also an integral ER membrane protein that does not require an N-terminal SP for ER targeting, but contains complex N- and C-terminal retention signals [33].

Upon deletion of the N-terminus (including the putative TMDs; Δ56), DHCR24 still associated with the membrane (Figure 3A), and could not be solubilized by various treatments (Figure 3B). Again, this is similar to cytochrome P450, which contains a hydrophobic N-terminus predicted to associate with the ER membrane, which retained strong membrane attachment after truncation experiments and differential solubilization [34,35]. Further N-terminal truncations in DHCR24 that deleted lower scoring TMD candidates also associated exclusively with the membrane (Figure 4). These data discount the findings of Lu et al. [9] that the N-terminus is essential for membrane attachment, and that deletion of the N-terminus (Δ58) results in altered cellular localization of DHCR24. In contrast, our findings demonstrate that the membrane association of DHCR24 is not reliant on TMDs at the N-terminus, but contains membrane associated region(s) beyond the hydrophobic N-terminus.

We also elucidated the membrane orientation of the N- and C-termini of DHCR24, for which the TMD prediction programs gave different results. Using protease protection assays, we determined that the C-terminus was cytoplasmic, due to its accessibility to trypsin. The N-terminus, however, was strongly protected, even when trypsin was accessible to the lumen by partial solubilization of the membrane (Figure 2A). This suggests that the N-terminus is ‘hidden’ from trypsin; either due to being embedded within the ER membrane, which would support (in part) Pedretti et al.'s ‘peduncle’ model [5], or due to peripheral interactions with the ER membrane. Both of these possible models disagree with the findings of Lu et al., that the N-terminus is lumenal, which is most likely due to the difference in epitopes used in the protease protection assay [9]. As mentioned, Lu et al. [9] used a large fusion protein (~28 kDa), whereas we used a small V5 epitope (~1 kDa). Testing the membrane association of a C-terminal truncation using a differential solubilization assay would determine the type of interaction the N-terminus has with the membrane (integral or peripheral). Finally, the identification of a digested N-terminal ~11 kDa fragment suggests that the membrane associated N-terminus is followed by a sterically hindered cytosolic loop at position ~70–100 (Figure 2B).

Overall, our findings demonstrate that DHCR24 has a strong affinity with the ER membrane, due to multiple membrane-associated regions, including beyond the N-terminus (Δ240; Figure 4). The extreme hydrophobic nature of the N-terminus suggests that it is also membrane associated, but this needs to be confirmed experimentally by C-terminal truncations. We hypothesize that there are multiple membrane associated regions throughout the protein, or re-entrant loops that associate with only one leaflet of the membrane. Re-entrant loops are typically rich in small residues such as glycines and alanines [36], and one of the lower scoring putative TMDs (d, Figure 1) is rich in these amino acids, and therefore a prime candidate for a re-entrant loop. Our proposed model may also contain classical TMD(s), as although this study ruled out the most likely putative TMD, one low-scoring putative TMD was untested (e, Figure 1). As this region is beyond the first 240 residues, it is likely to explain the strong membrane localization of Δ240 DHCR24 (Figure 4). Our data strongly suggest that relocation of DHCR24 to other cellular compartments, proposed to occur in response to stress signals, is highly unlikely [3]. This conclusion should be re-examined in light of our results.

**Figure 6:**
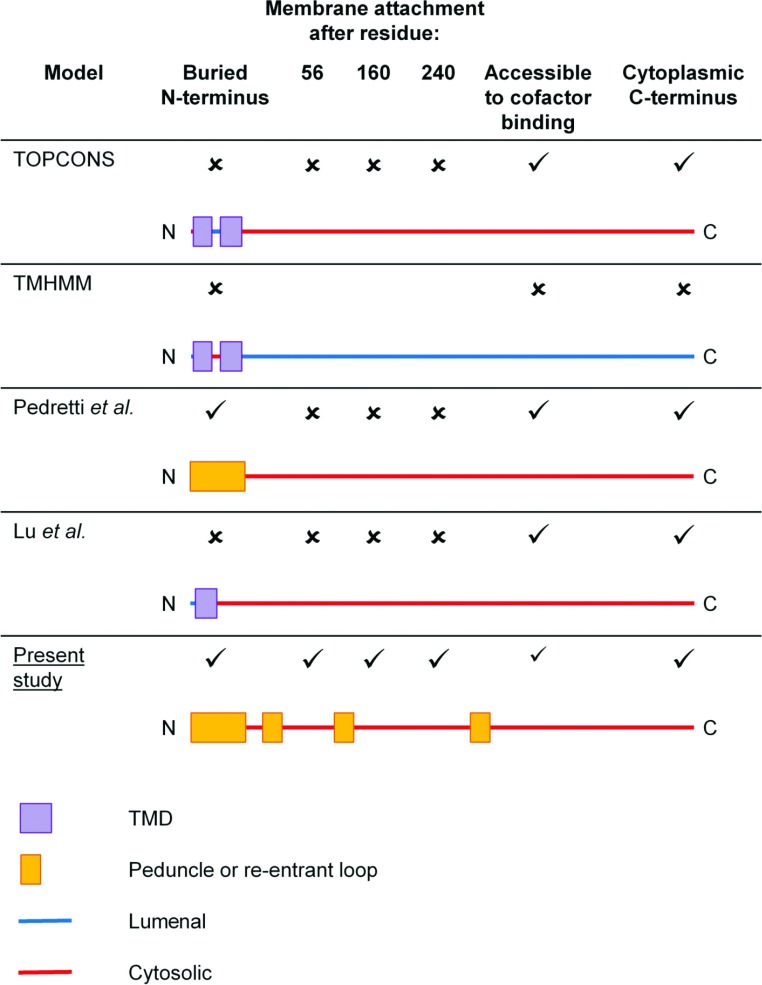
Comparison of current DHCR24 membrane topology models The predicted and published models for DHCR24 topology are presented with features indicated, compared with our hypothesized DHCR24 membrane topology model (the present study).

Altogether, our findings are at odds with prediction algorithms and published topology models (Figure 6, [5,9]). We demonstrated that DHCR24 does not conform to either published topology model; being neither monotopic, like Pedretti et al. suggest, nor bitopic, as Lu et al. propose, but is polytopic, with multiple hydrophobic domains passing through and/or anchoring DHCR24 to the hydrophobic core of the lipid bilayer of the ER.

**Figure 7:**
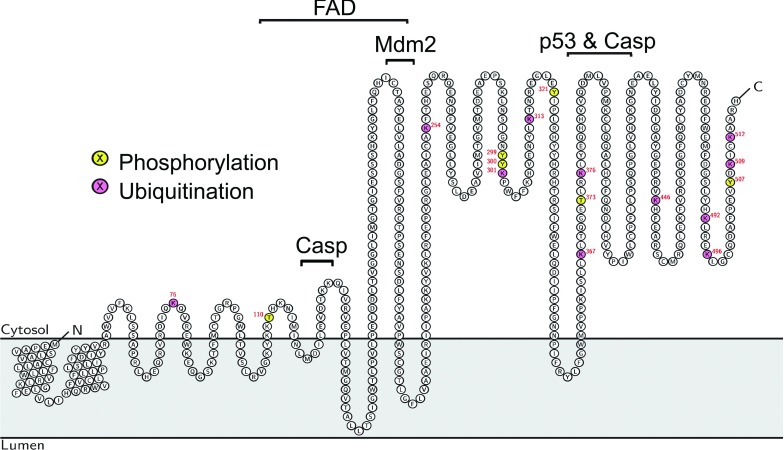
Hypothetical membrane topology model of DHCR24 This model integrates the known PTM sites to refine our working model of DHCR24 membrane topology. As the focus of this work has been on the N-terminus, this hypothetical model does not attempt to encapsulate the membrane topology of the C-terminus, which is likely to snorkel in and out of the membrane similar to the N-terminus, and is therefore protected from trypsin digestion (Figure 2B, lanes 1–4 *versus* 6–9). Cofactor-binding sites marked: FAD (111–203), p53 (358–425), Mdm2 (203–215). Caspase cleavage sites (122–127, 383–387).

To create a hypothetical membrane topology model of DHCR24, we have also utilized in addition to our experimental findings our knowledge of known PTMs on DHCR24 [37]. Phosphorylation and ubiquitination sites must be accessible to cytosolic kinases and ubiquitin ligases respectively, and therefore we have used these data to refine our topology model of DHCR24 (Figure 7). To the best of our knowledge, we are the first to propose that the expanding databases of PTMs provide an unlikely but invaluable resource for membrane topology mapping, including for other atypical cholesterogenic membrane proteins [38]. After all, this is not the first example of a cholesterol biosynthetic enzyme that has an atypical membrane topology. Lanosterol synthase [also known as OSC (oxidosqualene cyclase)] is a monotopic ER membrane protein, containing a membrane associated region, which resides in one leaflet of the membrane, and therefore does not span the bilayer [39]. This hydrophobic catalytic domain resides in the membrane, which allows access to the hydrophobic substrate, oxidosqualene. As the substrate-binding domain of DHCR24 is as yet uncharacterized, and desmosterol is also hydrophobic, a membrane associated catalytic site is likely.

To further refine our membrane topology model of DHCR24 (Figure 7), additional experimentation is required to precisely define membrane, cytoplasmic, and lumenal regions, including whether the final putative membrane region (e) is *bona fide*. This commonly requires modification of the specific residues or introduction of epitopes and recognition sites. Cysteine derivitization and glycosylation site mapping have previously been successful in elucidating the membrane topology of membrane bound proteins, such as Insig [13], and these techniques could similarly be applied here.

## Online data

Supplementary data
