# Supplementary material for: DHCR24 associates strongly with the endoplasmic reticulum beyond predicted membrane domains: implications for the activities of this multi-functional enzyme
Source: Biosci Rep. 2014 Mar 18;34(2):e00098. doi: 10.1042/BSR20130127 (PMC3958128; doi:10.1042/BSR20130127)
Supplement: Supplementary data [file bsr034e098add.pdf]

Biosci. Rep. (2014) / **34** / art:e00098 / doi 10.1042/BSR20130127

OPEN ACCESS

Original Paper

Bioscience Reports

www.bioscirep.org

Bioscience Reports

SUPPLEMENTARY DATA

DHCR24 associates strongly with the endoplasmic reticulum beyond predicted membrane domains: implications for the activities of this multi-functional enzyme

Eser J. ZERENTURK*, Laura J. SHARPE* and Andrew J. BROWN*[1]

*School of Biotechnology and Biomolecular Sciences, The University of New South Wales, Sydney, NSW 2052, Australia

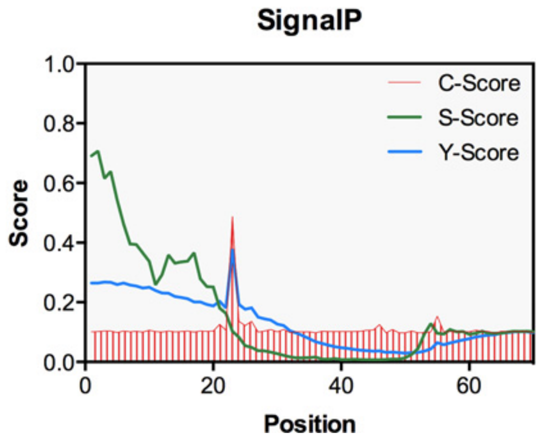

Figure S1 DHCR24 contains a putative signal sequence
Signal sequence prediction by SignalP v4.1, with the output given for the first 70 residues. C-score, raw cleavage site score; S-score, signal peptide score; Y-score, combined cleavage site score.

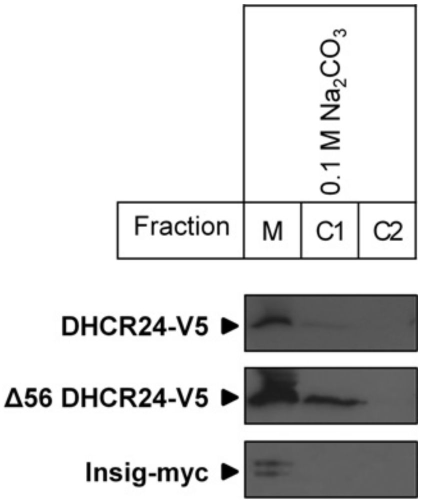

Figure S2 Membrane association of Δ56 DHCR24
As in Figure 3(B), CHO-7 cells were transfected with either 8 μg DHCR24-V5 or Δ56 DHCR24-V5, and co-transfected with 2 μg Insig-1-myc in a 14.5-cm dish for 24 h. Cell lysate was fractionated and membranes were isolated and resuspended in 0.1 M Na_2CO_3 pH 11.5 (high pH) and incubated for 30 min at 4 °C with end over end mixing, then ultra-centrifuged at 100 000 ***g***. The process was repeated using the supernatant (C1), with the resulting 100 000 ***g*** supernatant designated C2. C1, C2, and the pellet, representing the membrane (M) fraction were separated by SDS–PAGE (7.5 % gel) and immunoblotted with antibodies against V5 (DHCR24) and myc (Insig).

Received 29 November 2013/4 February 2014; accepted 5 February 2014

Published as Immediate Publication 6 February 2014, doi 10.1042/BSR20130127

[1] To whom correspondence should be addressed (email aj.brown@unsw.edu.au).

© 2014 The Author(s) This is an Open Access article distributed under the terms of the Creative Commons Attribution Licence (CC-BY) (http://creativecommons.org/licenses/by/3.0/) which permits unrestricted use, distribution and reproduction in any medium, provided the original work is properly cited.
